# Assessing the relationship between body image and quality of life among rural and urban breast cancer survivors in China

**DOI:** 10.1186/s12905-022-01635-y

**Published:** 2022-03-04

**Authors:** Jinghua An, Kaina Zhou, Minjie Li, Xiaomei Li

**Affiliations:** 1 School of Nursing, Health Science Center of Xi’an Jiaotong University, Yanta West Road 76, Xi’an, 710061 Shaanxi China; 2 Nethersole School of Nursing, The Chinese University of Hong Kong, Central Ave, Shatin, Hong Kong, SAR

**Keywords:** Body image, Quality of life, Breast cancer survivors, Rural population, Urban population

## Abstract

**Background:**

Breast cancer survivors experience altered body image and quality of life (QoL) due to the disease and its treatment. The multidimensional nature of body image and QoL makes their relationships complex. This study aimed to examine the associations between the two concepts in Chinese breast cancer survivors and test whether these associations are moderated by rural–urban residence.

**Methods:**

A cross-sectional design was adopted. Breast cancer survivors were recruited via a convenience sampling method. Two validated questionnaires (the Body Image Self-Rating Questionnaire for Breast Cancer and 36-item Short-Form Health Survey) and questions assessing demographic and clinical covariates were administered. Multiple linear regressions were used to assess the relationship between body image and QoL domains and to examine the moderating effect of rural–urban residence.

**Results:**

In our sample of 354 breast cancer survivors, half (50.28%) lived in rural areas. After adjusting for demographic and clinical variables, better perception of body image-related sexual activity change, role change, and psychological change was significantly associated with better physical (β ranged from − 0.15 to − 0.11, *p* < 0.05) and mental (β ranged from − 0.46 to − 0.34, *p* < 0.001) well-being. Better perception of body image-related social and behavior change was significantly associated only with better mental well-being (β ranged from − 0.40 to − 0.33, *p* < 0.001). The association between body image and mental well-being was much stronger in urban subjects (b = − 0.38, *p* < 0.001) than in rural subjects (b = − 0.20, *p* < 0.001).

**Conclusions:**

Our findings suggest that multidimensional body image is associated with physical and mental well-being in Chinese breast cancer survivors. Body image appears to play a larger role in urban breast cancer survivors’ mental well-being. Our results indicate that incorporating interventions that address body image issues would be advantageous for survivorship care to enhance QoL in breast cancer survivors. Furthermore, rural–urban differences should be considered in the strategic design of survivorship care programs in rural and urban settings.

## Background

In China, breast cancer is the most common cancer diagnosed among women and is the second-leading cause of cancer death in women [1]. Breast cancer surgery and adjuvant therapies can have many adverse impacts on women. These impacts can include changes to their physical appearance, such as loss of a breast, scarring, alopecia, and skin alterations as well as weight change. The physical appearance changes, together with symptoms such as sensation alteration, arm function impairment, and fatigue, can affect breast cancer survivors’ mental image of their bodies [2–4]. Body image encompasses one’s body-related self-perceptions, attitudes, and behaviors [5], and body image concerns are one of the most common psychosocial issues experienced by breast cancer survivors [3, 6, 7]. Poor body image has been linked to depression [6, 8], sexual concerns [9, 10], and psychological distress in breast cancer survivors [3, 7].

Helping breast cancer patients adapt to aesthetic and functional changes resulting from the disease and its treatment, whether temporary or permanent, has the potential to reduce body image-related distress and thus improve patients’ quality of life (QoL) [11, 12]. Thus, the nature of the relationships between major domains of body image and QoL need to be understood, as different aspects of body image may play different roles in influencing survivors’ QoL [11]. Researchers have reported that overall body image was correlated with QoL in breast cancer survivors [8, 13], but few have explored the relationships between the various body image and QoL domains. Moreover, no study has examined these relationships in Chinese breast cancer survivors, who are more likely to be diagnosed at a much younger age and more advanced stages than women in high-income countries [14]. Chinese breast cancer survivors also tend to undergo more aggressive treatments and are less likely to have breast reconstruction; a reconstruction rate of only 3.4–4.5% was observed among Chinese survivors [15, 16]. Reported potential reasons for the low reconstruction rate included Chinese women’s smaller breast volume, their lack of awareness of breast reconstruction, a shortage of skilled surgeons, lack of cooperation between oncology and plastic surgery departments, and lack of insurance coverage [16–18].

In addition, little research attention has been given to body image among rural breast cancer survivors. In China, rural survivors tend to exhibit more aggressive breast cancer than urban survivors, leading to a higher proportion of modified radical mastectomies and more adjuvant treatments in rural survivors [19]. The relationship between body image and QoL likely differs due to various determinants of rural–urban disparities [20]. However, no previous research has examined rural–urban residence as a moderator of the relationship between body image and QoL in breast cancer survivors. Understanding the role of place of residence may support strategic design of care plans for breast cancer survivors in urban and rural settings.

**Fig. 1:**
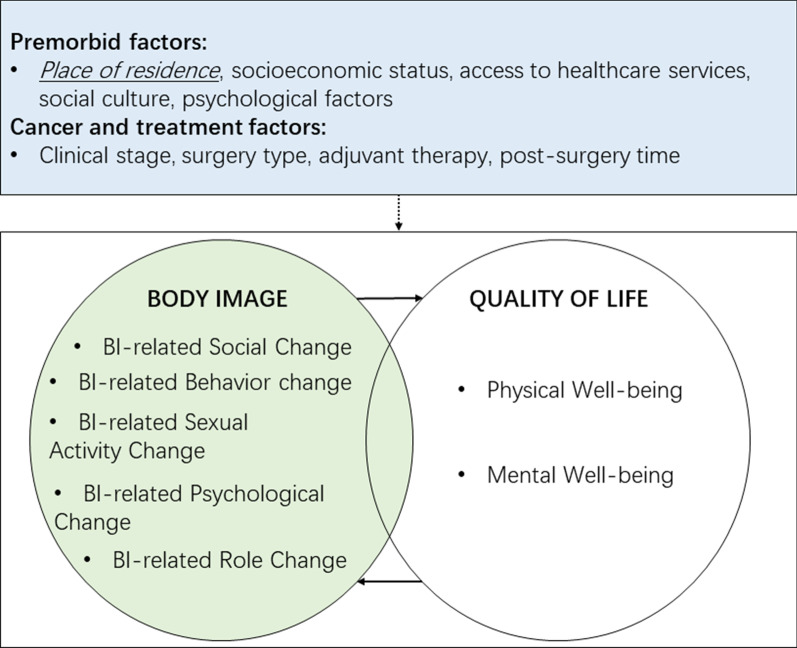
Conceptual framework of associations between body image and quality of life in breast cancer survivors

Figure 1 depicts our conceptual framework regarding potential associations between body image and QoL domains. It was derived from a literature review of the two concepts of interest—QoL [21, 22] and body image [5, 23]—and theoretical frameworks addressing relationships between body image and QoL in different medical contexts [11, 12]. The multifaceted construct of body image not only addresses how a woman views her appearance and femininity, but also encompasses body image-related changes in her roles (in conducting daily activities and functioning as a mother and worker), her social involvement, her behaviors (such as avoiding attention to her body and repeatedly checking her breast), and her sexual functioning [4]. Also, we focus on two major QoL domains that body image may affect: physical and mental well-being. Physical well-being encompasses one’s perceived physical function, bodily pain, and role limitations due to physical problems [22, 24]. Mental well-being mainly involves social functioning, emotional and mental health, and role limitations due to emotional problems [22, 24, 25].

We posit that different domains of body image-related changes are correlated with mental and physical well-being in breast cancer survivors. The constructs of body image and QoL affect each other and overlap from a conceptual standpoint, but each has unique features [11]. Based on the literature, other factors also influence breast cancer survivors’ body image and QoL [2, 11, 26]. One broad category, premorbid influences, represents past experience and one’s position in society [11, 26], including factors such as place of residence and access to healthcare services. Another category is the cancer itself and its treatment factors [2, 11, 26]. In this study, we define place of residence in terms of self-identification because this is especially useful when focusing on individual subjective experience such as body image and QoL, which are influenced by self-identity as well as cultural perceptions. Also, we consider women to be breast cancer survivors from the time of diagnosis until the end of life [27].

Based on our conceptual framework, this study aimed to (1) examine associations between body image and QoL domains in Chinese breast cancer survivors and (2) test whether the associations are moderated by place of residence. Our findings will enhance understanding of rural–urban disparities in biopsychosocial health and will inform development of evidence-based clinical practice to address the body image concerns of breast cancer survivors and improve their overall QoL.

## Methods

### Study design

A cross-sectional, associational design was adopted.

### Study setting and sample

Data were collected in the Shaanxi Tumor Hospital and First Affiliated Hospital of Xi'an Jiaotong University in Xi’an, a large city in northwestern China. Due to the high concentration of healthcare resources in China’s cities, rural residents almost always travel to larger hospitals in urban areas to obtain cancer diagnosis and treatment. Hospitals in rural regions and small towns typically do not have the equipment or skilled staff needed to diagnose or treat major conditions such as breast cancer.

A convenience sample of 354 subjects was recruited. The nurses in the Breast Surgery Departments provided a list of inpatients who had been diagnosed with breast cancer and undergone breast cancer surgery. The researchers then approached listed inpatients individually to explain the study details, screen them for eligibility, and invite eligible individuals to participate. All subjects were aged 18 years or older, were female, had been pathologically diagnosed with breast cancer, and had undergone breast cancer surgery. Women with metastatic breast cancer or having been diagnosed with other cancers were excluded. Women who provided informed consent to participate completed the research survey and returned it to trained researchers who remained in the hospitals during the data collection period. Subjects with reading or writing difficulties were interviewed by the researchers to record their responses.

### Measures

#### Demographic and clinical covariates

Using a questionnaire prepared by the researchers, subjects provided information on their demographics (e.g., place of residence and educational attainment) and clinical treatment (e.g., clinical stages and surgery types). Unlike many studies that defined place of residence in terms of subjects’ *Hukou* (their registered household of origin in rural or urban areas) [28], we defined place of residence as the subjects’ reported main residence during the past 3 months; this operationalization reflects current place of residence, which is often inconsistent with *Hukou* registrations. The residence options were rural (agricultural areas) and urban areas (cities), defined by the National Bureau of Statistics [29].

#### Body image

Body image domains were measured with the Body Image Self-Rating Questionnaire for Breast Cancer (BISQ-BC) developed by Zhou et al. [4]. This instrument was developed specifically for use with breast cancer patients in China. Also, it highlights key aspects of changes related to body image, which serves to enhance understanding of the process of psychological adjustment to bodily changes. The BISQ-BC includes five subscales: body image (BI)-related behavior change, BI-related sexual activity change, BI-related role change, BI-related psychological change, and BI-related social change. The questionnaire employs a 5-point Likert scale; responses to each item range from 1 (strongly disagree) to 5 (strongly agree). For the subscales and the scale as a single measure, higher scores indicate poorer body image. In this study, the Cronbach’s alpha values for the five subscales ranged from 0.72 to 0.82.

#### Quality of life

QoL domains were assessed with the Chinese version of the (SF-36 v2.0) [25]. It is a multidimensional questionnaire consisting of eight subscales: physical function, role-physical, bodily pain, general health, vitality, social function, role-emotional, and mental health. These subscales are used to calculate the physical component summary (PCS) and mental component summary (MCS). Health Outcomes Scoring Software 2.0 (QualityMetric Incorporated) was used to calculate the eight subscale scores and two summary components [25]. In our conceptual framework, physical well-being and mental well-being were defined as the PCS and MCS of the SF-36. Higher scores of PCS and MCS indicate better QoL. The Cronbach’s alpha values for the eight subscales ranged from 0.73 to 0.90 in this study.

### Statistical analyses

The two-sample t-test and chi-square test were used to compare demographic and clinical characteristics of subjects currently living in rural and urban areas. Descriptive statistics were calculated for QoL and body image, and comparisons were made between rural and urban subjects employing the two-sample t-test. Multiple linear regressions were subsequently conducted to assess the relationship between body image subscales and the two QoL summary components after adjusting for covariates.

Next, to test the moderating effect of place of residence, we followed the procedures outlined by Aiken and West for testing interactions between a continuous independent variable and a categorical moderator [30]. After controlling for demographic and clinical covariates in hierarchical linear regressions, the body image total score and place of residence (reference: rural areas) were entered in Step 1, and the interaction term (place of residence × body image) was entered in Step 2 to test the moderation effect and yield the changed R square. The predictors were centered around 0 to reduce multicollinearity. Post-hoc simple slope analyses were conducted to determine the nature of the significant interactions.

## Results

Table 1 depicts demographic and clinical covariates for the study subjects. Overall, subjects’ mean age was 49.65 years (SD = 9.83). Most subjects were married (95.76%), with an education level of secondary school or less (78.81%), and a monthly household income per capita over the past year (monthly income) of less than 3000 yuan (68.27%). Half of the subjects (*n* = 178, 50.28%) lived in rural areas. Rural subjects had significantly lower educational attainment; only 2.81% had tertiary education, compared with 39.77% of their urban counterparts. Rural subjects also had lower monthly income; 10.67% of rural subjects were paid > 3000 yuan, compared with more than half of the urban subjects. At the time of our research, 58.99% of the rural women were unemployed compared to only 21.02% of the urban women. As to their clinical characteristics, rural and urban subjects differed significantly in their surgery types. Rural subjects tended to have undergone more aggressive surgeries, with 69.10% having modified radical mastectomy versus 57.39% of urban subjects.

**Table 1:** Demographic and clinical characteristics of participants^a^ (*n* = 354)

Characteristics	Rural participants	Urban participants	Statistics (t/χ^2^)	*p* value
Demographics				
Years of age, mean (SD)	49.04 (9.56)	50.26 (10.09)	− 1.17	0.88
Educational attainment, N (%)			**117.46**	**< 0.001**
Primary and lower	62 (34.83)	11 (6.25)		
Secondary	111 (62,36)	95 (53.98)		
Tertiary	5 (2.81)	70 (39.77)		
Marital status, N (%)			0.06	0.81
Married	170 (95.51)	169 (96.02)		
Other	8 (4.49)	7 (3.98)		
Monthly household income per capita over the past year (Chinese Yuan), N (%)			**98.37**	**< 0.001**
< 1000	76 (42.70)	11 (6.25)		
1000–3000	83 (46.63)	71 (40.34)		
> 3000	19 (10.67)	93 (52.84)		
Employment status, N (%)			**89.98**	**< 0.001**
Employed	66 (37.08)	63 (35.80)		
Unemployed	105 (58.99)	37 (21.02)		
Retired	7 (3.93)	76 (43.18)		
If employed, occupation			**69.41**	**< 0.001**
Peasants	51 (77.27)	4 (6.35)		
Self-employed	5 (7.58)	5 (7.94)		
Elementary Laborers	6 (9.09)	5 (7.94)		
Professionals/managers	1 (1.52)	31 (49.21)		
Others	3 (3.55)	18 (28.57)		
Clinical characteristics				
Surgery type, N (%)			**6.13**	**0.047**
Modified radical mastectomy	123 (69.10)	101 (57.39)		
Total mastectomy	43 (24.16)	53 (30.11)		
Lumpectomy	12 (6.74)	22 (12.50)		
Chemotherapy, N (%)			0.96	0.62
Undergoing	136 (76.40)	133 (75.57)		
Completed	37 (20.79)	35 (19.89)		
No chemotherapy	5 (2.81)	8 (4.55)		
Clinical stage, N (%)			3.06	0.22
0 and I	29 (16.29)	42 (23.86)		
II	102 (57.30)	91 (51.70)		
III and IV	47 (26.40)	43 (24.43)		
Post-surgery time (months), mean (SD)	2.80 (2.75)	3.18 (3.40)	− 1.15	0.87

*SD* standard deviation

^a^Significant associations/differences (*p* < 0.05) are shown in bold

Table 2 summarizes the scoring of the eight subscales and two summary components (PCS and MCS) for QoL, the five subscales for body image, and the body image total score. Urban subjects had better mental well-being than rural subjects, while their physical well-being did not differ significantly. Urban subjects had significantly better overall body image. They also had lower body image scores for four of the five domains, and the differences were statistically significant for BI-related role change and BI-related psychological change.

**Table 2:** Body image and quality of life^a^ (*n* = 354)

	Overall (Mean ± SD)	Rural (Mean ± SD)	Urban (Mean ± SD)	MD (95% CI)	*p* value
Physical function	45.33 ± 6.47	44.77 ± 6.75	45.68 ± 6.12	− 0.91 (− 2.26, 0.43)	0.19
Role limitations due to physical problems	34.91 ± 9.42	34.03 ± 8.35	35.49 ± 10.12	− 1.46 (− 3.40, 0.48)	0.14
Bodily pain	47.64 ± 10.11	47.79 ± 10.21	47.14 ± 9.98	0.64 (− 1.47, 2.76)	0.55
General health	43.06 ± 9.33	*42.04* ± *8.60*	*43.79* ± *9.74*	− *1.74 *(− *3.66, 0.18*)	*0.07*
Vitality	48.75 ± 8.85	*47.81* ± *8.64*	*49.41* ± *8.94*	− *1.60 *(− *3.44, 0.24*)	*0.09*
Social functioning	39.04 ± 10.77	38.72 ± 10.28	39.11 ± 11.08	− 0.39 (− 2.62, 1.85)	0.73
Role limitations due to emotional problems	38.80 ± 10.83	*37.61* ± *10.31*	*39.73* ± *11.27*	− *2.12 *(− *4.38, 0.13*)	*0.07*
Mental health	44.47 ± 8.77	**43.24 ± 8.54**	**45.52 ± 8.90**	− **2.28 (**− **4.10, **− **0.45)**	**0.01**
Physical component summary	43.66 ± 6.52	43.38 ± 6.05	43.64 ± 6.82	− 0.26 (− 1.61, 1.08)	0.70
Mental component summary	42.49 ± 9.49	**41.30 ± 9.06**	**43.46 ± 9.79**	− **2.15 (**− **4.13, **− **0.18)**	**0.03**
BI-related social change	6.17 ± 1.89	6.35 ± 1.80	6.05 ± 1.96	0.30 (− 0.09, 0.70)	0.13
BI-related behavior change	24.61 ± 4.05	24.63 ± 3.98	24.67 ± 4.13	− 0.04 (− 0.89, 0.81)	0.92
BI-related sexual activity change	12.36 ± 2.60	12.45 ± 2.51	12.31 ± 2.72	0.15 (− 0.40, 0.69)	0.60
BI-related role change	14.77 ± 3.37	**15.44 ± 3.17**	**14.21 ± 3.44**	**1.24 (0.54, 1.93)**	**< 0.01**
BI-related psychological change	25.20 ± 5.27	**26.03 ± 4.76**	**24.58 ± 5.61**	**1.45 (0.37, 2.54)**	**< 0.01**
BI summary	83.17 ± 14.42	**84.95 ± 13.24**	**81.87 ± 15.30**	**3.08 (0.09, 6.07)**	**0.04**

*BI* body image, *MD* mean difference, *SD* standard deviation, *95% CI* 95% confidence interval

^a^Significant differences (*p* < 0.05) are marked in bold. Marginally significant differences (*p* < 0.1) are italicized

Table 3 shows the relationships between the five body image domains and two QoL components. Through analysis of adjusted regression models, the five body image domains were significantly associated with mental well-being after controlling for demographic and clinical covariates, with standardized coefficients ranging from − 0.46 to − 0.33. Also, three body image domains (BI-related sexual activity change, BI-related role change, and BI-related psychological change) were significantly associated with physical well-being after controlling for covariates; the standardized coefficients were smaller, ranging from − 0.15 to − 0.11. On the whole, breast cancer survivors with a better perception of their body image reported greater mental and physical well-being.

**Table 3:** Adjusted multiple linear regression models assessing relationships between body image and quality of life domains^a,b^ (*n* = 354)

	PCS			MCS		
	Std. β	95% CI	*p* value	Std. β	95% CI	*p* value
BI-related social change	− 0.07	− 0.16, 0.02	0.20	− **0.40**	− **0.49, **− **0.32**	**< 0.001**
BI-related behavior change	− 0.09	− 0.18, 0.00	0.11	− **0.33**	− **0.41, **− **0.24**	**< 0.001**
BI-related sexual activity change	− **0.11**	− **0.21, **− **0.03**	**0.03**	− **0.34**	− **0.42, **− **0.25**	**< 0.001**
BI-related role change	− **0.14**	− **0.23, **− **0.05**	**0.01**	− **0.38**	− **0.47, **− **0.30**	**< 0.001**
BI-related psychological change	− **0.15**	− **0.24, **− **0.06**	**0.01**	− **0.46**	− **0.54, **− **0.38**	**< 0.001**

*BI* body image, *MCS* mental component summary, *PCS* physical component summary, *Std. β* standard coefficient, *95% CI* 95% confidence interval

^a^All linear regression models were adjusted for the following covariates: age, education attainment, marital status (ref: married), reside (ref: rural areas), monthly income, employment status (ref: employed), clinical stage (ref: stage 0 and I), surgery type (ref: modified radical mastectomy), adjuvant chemotherapy status (ref: undergoing chemotherapy), and post-surgery time

^b^Significant associations (*p* < 0.05) are marked in bold

**Fig. 2:**
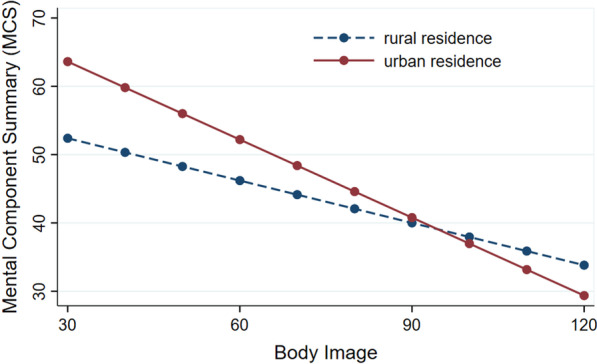
Moderation effects of rural–urban residence on associations between body image and mental well-being (N = 354)

Table 4 summarized the test of the moderation effect of place of residence. In hierarchical linear regression, the addition of the interaction term at Step 2 explained an additional 1.66% of the variance (*p* = 0.007) in mental well-being. Post-hoc slope analyses demonstrated that the association between body image and mental well-being was much stronger in urban subjects (b = − 0.38, *p* < 0.001) than in rural subjects (b = − 0.20, *p* < 0.001). Figure 2 displays this moderation effect of place of residence. In explaining physical well-being, no significant interaction between body image and place of residence was found in Step 2.

**Table 4:** Hierarchical linear regression testing the interaction effects of place of residence^a,b^ (*n* = 354)

	PCS					MCS				
	Std. β	95% CI	*p value*	ΔR^2^	adjR^2^_cum_	Std. β	95% CI	*p value*	ΔR^2^	adjR^2^_cum_
Step 1				**0.10**	**0.06**				**0.24**	**0.21**
BI	− **0.14**	− **0.23, **− **0.05**	**0.009**			− **0.46**	− **0.53, **− **0.38**	**< 0.001**		
Place of residence (Ref: rural areas)	0.08	− 0.20, 0.05	0.32			0.10	− 0.21, 0.02	0.17		
Step 2				0.003	**0.06**				**0.02**	**0.23**
BI	− 0.08	− 0.16, 0.003	0.34			− **0.31**	− **0.44, **− **0.19**	**< 0.001**		
Place of residence (Ref: rural areas)	0.08	− 0.05, 0.20	0.30			0.11	− 0.009, − 0.22	0.13		
Place of residence × BI	− 0.09	− 0.22, 0.05	0.28			− **0.20**	− **0.32, **− **0.08**	**0.007**		

*BI* body image, *MCS* mental component summary, *PCS* physical component summary, *Std. β* standard coefficient, *95% CI* 95% confidence interval

^a^All linear regression models were adjusted for the following covariates: age, education attainment, marital status (ref: married), monthly income, employment status (ref: employed), clinical stage (ref: stage 0 and I), surgery type (ref: modified radical mastectomy), adjuvant chemotherapy status (ref: undergoing chemotherapy), and post-surgery time

^b^Regression models with *p* < 0.05 and significant coefficients (*p* < 0.05) are marked in bold

## Discussion

Our study focused on rural and urban Chinese breast cancer survivors whose body image and its associations with QoL have not received adequate attention in research and practice. After controlling for demographic and clinical covariates, having better body image in any of the five domains (BI-related social change, behavior change, sexual activity change, role change, and psychological change) was associated with better mental well-being. The close correlation between all 5 domains of body image and mental well-being was supported by past research. Breast cancer survivors with poorer body image typically had higher levels of anxiety and depression [6, 8, 31]. A meta-synthesis concluded that breast cancer survivors’ perception of physical appearance changes triggered discrepancy between self and societal image of femininity, leading to altered identity; women who were unable to renegotiate or reposition their identity experience emotional distress [7]. Also, a longitudinal study in breast cancer survivors found that body image-related psychological changes (fear of recurrence and death, lower satisfaction with physical appearance) predicted negative affections (unpleasantness and subjective distress such as anger and sadness) 6 months after mastectomy [31]. Our study indicated that improving all subdomains of body image is key to promoting mental well-being in breast cancer survivors.

Among the five domains of body image, poorer perceptions of BI-related sexual activity changes, role changes, and psychological changes were associated with decreased physical well-being, indicating interactions between mind and body. As the possible mechanism involved, failing to adapt to altered body image may have resulted in psychological distress and chronic stress, which were shown in research to cause cancer patients’ fatigue, impaired sleep, and cognitive dysfunction by affecting the neuroendocrine system and inducing inflammation [32]. This mind–body interaction has received much research attention in cancer survivors; lower positive psychosocial factors (e.g., less social support) and higher negative psychosocial factors (e.g., higher anxiety and perceived stress) were associated with biomarkers indicating worse clinical outcomes (e.g., chronic elevations of cortisol) [33, 34]. Our findings align with our conceptual framework in that they affirm the correlations between multiple body image and QoL domains. Prospective research is warranted to further investigate the potential bidirectional associations between body image and QoL.

Our study framework and findings suggest that there may be some universal benefits of improving positive perception of body image. To improve Chinese breast cancer survivors’ body image, access to lumpectomy and reconstruction needs to be increased. Survivors with lumpectomy were shown to experience better overall body image than those with mastectomy [26, 35–37]. Reconstruction also contributes to women’s wholeness and addresses their body image concerns to some extent [36, 38]. The advantages and disadvantages of these surgery options should be discussed with Chinese breast cancer patients to assist informed decision-making on surgeries. Also, in line with a review on managing body image in adult cancer patients [39], healthcare professionals are recommended to discuss body image with every breast cancer survivor and refer them to a mental health specialist if needed. In addition, interventions that assist in adapting to body image-related changes need to be developed and integrated into survivorship care [40].

Regarding the moderation effect of rural–urban residence, body image appears to be more important to urban women than rural women. The mechanism of how place of residence moderates the association is beyond the scope of this study. Yet there are three potential explanations. First, rural breast cancer survivors may have health needs or concerns more important than body image. They were reported to have many more unmet needs for healthcare system information and more limited access to supportive care than their urban counterparts [41]. Thus, rural survivors may have to prioritize their healthcare system information needs and health service needs, with body image becoming less important for them even if they have significantly worse body image and mental well-being than urban survivors.

Second, rural breast cancer survivors in China may focus more on practical livelihood issues such as financial needs and impaired ability to perform manual labor**,** rather than body image. In our study, only 10.67% of rural subjects had a monthly income of over 3000 yuan, compared with over half of urban subjects who were earning this much. Rural subjects were thus more likely to experience financial toxicity than urban subjects. In addition, among subjects who were employed at the time of our study, 77.27% of rural subjects were peasants, whereas very few urban subjects (6.35%) were peasants; only 3.93% of rural subjects were retired, compared with 43.18% of urban subjects. In China, peasants mostly reside in rural areas and usually do not “retire” at a specific age. Thus, such subjects would have continued to work and produce wealth for their family if they had not been diagnosed with breast cancer. Similar to our finding of more rural subjects being unemployed than urban subjects (58.99% versus 21.02%), a recent study reported that rural Chinese cancer survivors were at higher risk for stopping work after treatment compared with urban Chinese and rural American cancer survivors; 40% of rural Chinese cancer survivors reported stopping work or farming due to cancer, and 39% reported reducing working hours [42].

Finally, urban breast cancer survivors may put more effort into addressing their body image-related concerns and cope with them more effectively. Our urban subjects had a populous living environment and their occupations typically involved less manual labor but more interaction with people; for example, 49.21% of urban employed subjects were professionals/managers, compared with 1.52% of rural subjects. Therefore, urban survivors being diagnosed and treated for breast cancer usually impaired their working ability less, and they might have valued their body image more due to frequent social contact. A study in Australian adult women showed that engagement in more social interactions was predictive of subsequently improved body satisfaction [43]. In addition, previous research [44] demonstrated that living in rural China and having less education predicted less adaptive coping patterns in breast cancer patients. Thus, rural breast cancer survivors may cope with body image less effectively.

Given the results presented in this article, it is important to recognize some limitations. First, our sample was convenience-based and was recruited in only two hospitals, which limited the generalizability of our findings. However, the two tertiary hospitals serve populations living in Shaanxi and nearby provinces, so our subjects were likely representative of Chinese breast cancer survivors living in northwestern China, where the economy is less developed than in coastal areas. In addition, we did not consider the influence of migrant-worker status. Many rural women migrate to urban areas to access more job opportunities in rapid urbanizing China. As a result, they are geographically closer to quality healthcare resources, but face new barriers to healthcare, such as reimbursement for health services [19, 45]. It is unclear how migration influences the associations between body image and QoL among women with breast cancer.

## Conclusions

Based on previous theory and research, we developed a conceptual framework to better understand body image and QoL in breast cancer survivors. Among our findings, Chinese breast cancer survivors with better body image reported greater mental and physical well-being. Our results indicate that incorporation of interventions that address body image issues would be advantageous for survivorship care programs focused on enhancing QoL in Chinese breast cancer survivors. In addition, body image appears to play a larger role in urban breast cancer survivors’ mental well-being. Further research is called for to uncover the underlying reason for the moderation effect because this is essential for strategically improving these survivors’ well-being as well as for promoting policy development for cancer care. Also, to fulfill their key role in educating breast cancer survivors and providing personalized care plans, healthcare professionals should consider the moderating role played by rural–urban residence when assessing and intervening in body image.

## Data Availability

The datasets used in the current study are available from the corresponding author on reasonable request.

## Abbreviations

QoL: Quality of life
BISQ-BC: Body Image Self-Rating Questionnaire for Breast Cancer
PCS: Physical component summary
MCS: Mental component summary
